# “I am in other people's hands as regards my health” A sociological critique of health care encounters of people with cirrhosis. A secondary analysis

**DOI:** 10.1177/17423953211058422

**Published:** 2021-11-23

**Authors:** Sarah Davis, Paul Higgs, Louise Jones, Lynda Greenslade, Jo Wilson, Joseph TS Low, Principal Research Fellow

**Affiliations:** 1 Marie Curie Palliative Care Research Department, Division of Psychiatry, University College London, London, UK; 2 Division of Psychiatry, University College London, London, UK; 3 Sheila Sherlock Liver Unit, Royal Free Hospital, London, UK; 4 Department of Palliative Care, 4965 Royal Free London NHS Foundation Trust, London, UK

**Keywords:** Cirrhosis, medical encounters, medical power, doctor patient interaction, patient experience

## Abstract

**Objectives:**

People with cirrhosis are encouraged to participate in shared decision-making with their doctors, but studies suggest that doctors limit the amount of information that is shared. In this study we explore the presence of medical power in clinical encounters in 2015 from a patient perspective and highlight its effects on healthcare interactions.

**Methods:**

Qualitative semi-structured interviews were conducted with ten people with cirrhosis attending a tertiary liver transplant centre in southern England. We explored their understanding of their disease and prognosis, and their participation in decision-making. Using the lens of medical power as a framework, we analysed findings into thematic sentences to summarise key ideas whilst preserving the complexity of identified concepts.

**Results:**

Three key concepts explained patient perspectives of their communication with doctors: (1) portraying a positive image to doctors, (2) avoiding confrontation with doctors, (3) feeling powerless in the face of doctors’ medical knowledge. These concepts show deeper dynamic issues of power during healthcare encounters, illustrated by participants’ reluctance to voice their concerns and express themselves, challenge decisions, or seek information.

**Conclusion:**

People with cirrhosis struggle to articulate their concerns or challenge decisions on their care and treatment and may worry about potential consequences. Our findings demonstrate the continuing persistence of issues of power at play in contemporary health care.

## Introduction

Advanced chronic liver disease (cirrhosis) is a complex condition with increased symptoms during exacerbations and prognostic uncertainty. It is the major cause of premature death in the UK and its incidence is rising due to increased alcohol consumption, viral hepatitis and obesity.^1^ Complications of cirrhosis are frequent and often require hospital admission.^2^ Liver transplants are the curative treatment option, but the majority of people with cirrhosis are clinically unsuitable for or unable to access a transplant and they face deteriorating health and a terminal prognosis. Of those people selected for transplant, 20% die before a compatible organ becomes available.^3^ People with cirrhosis often experience a range of burdensome physical and psychological symptoms, financial problems and social isolation.^4^ Many might benefit from an active palliative approach to care, aiming to improve their quality of life by addressing any physical, emotional and spiritual needs, and to support their families.^5^ Such an approach remains relevant for people awaiting liver transplant (LT) who are often mentally distressed and face uncertainty around whether a potentially life-saving donor may become available.^6,7^ Advanced liver disease has many causes, not all related to alcohol consumption. People may feel stigmatised even if their illness is not related to alcohol or substance misuse.^8–10^ For those where alcohol intake or addiction are relevant, there may be additional co-existing physical health, mental health and social problems.

Management of cirrhosis is challenged by the uncertainty of the disease trajectory, limited options for successful management, physical and cognitive insufficiency, communication barriers sometimes in the context of social isolation, and the curative focus of treatment.^2,4^ These challenges may affect the information that doctors feel able to discuss with people with cirrhosis and those close to them. Current research indicates that patients feel poorly informed about their condition and its likely progression and are unable to seek information and plan for future care.^11^ Doctors are aware that patients and family members often have little understanding of their disease or its severity.^12^ Professionals report lacking both the skills and confidence to have discussions regarding causes, treatments and outcomes early in advanced liver disease, consequently difficult conversations are delayed until the terminal stages, often bypassing patients themselves and being conducted solely with family members.^2^

Previous studies^2–4,11^ have highlighted the problem of communication in cirrhosis, and in this analysis we use a sociological lens to examine healthcare encounters. We aim to understand the hierarchical structures that may affect communication in the doctor-patient relationship. We do not criticise the work of doctors who endeavour to provide highly skilled compassionate care in a pressurised environment, rather we attempt to understand the impact of medical power in healthcare encounters. By medical power we refer to the sociological concept fusing the implicit imbalance in knowledge of the patient about their condition with a corresponding control over resources by the medical practitioner in medical encounters.^13^ Power in this sense is seen to be located in the process of occupational control that is part of the professionalisation process developed by medicine over the past century and a half;^14^ an institutional arrangement which creates an asymmetry between doctor and patient. Following the work of the American sociologist Talcott Parsons, the position of the patient is legitimated, through their adoption of the ‘sick role’ and by the doctor's corresponding adoption of the ‘physician role’.^15^ The differential power that each participant has is clearly acknowledged and is reflective of the less equal society of mid-century America^16,17^ and has therefore been seen as an inappropriate model, endorsing as it seems a less than equal relationship between doctor and patient.

Current guidelines reflect uneasiness with implicit unequal power relations and emphasize the importance of improving the communication skills of all health professionals to facilitate meaningful sharing of information.^18^ Contemporary health care has seen a shift from medical paternalism, defined by the interference of doctors with patients’ ability to make a choice regarding their healthcare,^19^ to a less hierarchical relationship in which the views and role of the person, who has a disease and is unwell and seeks advice or care, are given central consideration.^20,21^ Patients expect (and are expected) to be fully informed and involved in all aspects of their treatment.^22,23^ This is counter to Parsons idea of the ‘sick role’^15^ and its implicit acceptance of medical power. Under contemporary approaches, patients have the right to be informed by their doctors of all the relevant information and options for treatment regarding their condition when making decisions. Equally, doctors are expected to encourage patients to become more active and knowledgeable in treatment decisions,^22^ thereby democratising unequal relations in health care encounters.

Critical to this movement is the embedding of ‘shared decision-making’ in healthcare wherein the doctor-patient relationship is construed as a continuing partnership.^24^ Shared decision-making allows both doctor and patient to have joint responsibility regarding the sharing of medical information and the choice of treatment options.^25^

The impact of these policies, and more broad social changes, might suggest that encounters between doctors and patients are now more equal. However, as several commentators have observed,^26–28^ the dynamics between doctors and patients can reflect a more paternalistic relationship in which the concepts of unequal power remain.^29^ Challenges to shared decision-making are observed among people with liver disease. Perceived self-stigma connected to a diagnosis of cirrhosis is associated with adverse attitudes and decreased healthcare-seeking behaviour.^10^ Self-stigma may be stronger amongst those whose cirrhosis is related to substance abuse, rendering them less assertive in the doctor-patient relationship; people may blame themselves or think they are undeserving of care..^30,31^

We conducted a larger study of palliative care provision for people with cirrhosis.^2^ Our analysis explored patients’ perspectives and experiences of healthcare in a liver transplant tertiary centre. We focused on people’s understanding of their illness, prognosis and plans for future care, the nature of their communication with doctors, and how and when they participate in treatment decisions. During our analyses, we became aware of examples of medical power in health care encounters. In this paper, we report a secondary analysis, arising from our wider data, of the impact of medical power on doctor-patient interaction from the patient perspective.

## Methods

### Study design

Qualitative descriptive study. A secondary analysis of patient interview data arising from a larger mixed methods study reported elsewhere.^2^ We have followed the Standards for Reporting Qualitative Research (SRQR).^32^ (see Appendix II)

### Participant recruitment

We recruited from out-patient clinics and the day treatment unit at a tertiary liver centre in Southern England between November 2014−February 2015. Potential participants were diagnosed with cirrhosis and had experienced at least one episode of decompensation. The research nurse (SD), with 5 years of experience conducting qualitative research, liaised with liver clinicians who identified eligible patients. SD then introduced and discussed the study with potential participants and obtained written informed consent from those willing and able to take part.

Liaising with clinicians, we selected a purposeful sample based on gender, cause of cirrhosis, and age. We approached 12 patients, of whom 5 males and 5 females agreed to take part; their mean age was 59 years (range 46−77 years) and the causes of their cirrhosis were various. Due to local demographics, our sample was predominantly white British (9/10). Five participants were awaiting a liver transplant and five were considered ineligible for transplant due to continued alcohol misuse or frailty (Table 1).

**Table 1.** Participant demographics.

ID	M/F	Age	Diagnosis	Transplant list
A	F	61	ARLD	x
B	F	77	PBC	x
C	M	58	ARLD	√
D	F	55	ARLD	x
E	M	67	PSC	√
F	F	46	AIH	√
G	M	56	HEP C	√
H	F	69	AIH	x
I	M	52	NASH	x
J	M	58	HEP C + ARLD + HCC	√

### Data collection

The research nurse (SD) interviewed participants in their place of choice (4 in a liver out-patient clinic; 4 in participants’ homes; 2 in a day treatment unit). We used semi-structured interviews to explore participants’ understanding of their disease from diagnosis to the present, their experiences of healthcare and their perceptions of possible future care and outcomes. Interviews were audio-recorded using a digital voice recorder and lasted for 28–62 min (median: 58 min) (Appendix I – topic guide). SD transcribed the interviews of all participants, ensuring that all personal data was pseudonymised.

### Data analysis

Initially, we used a Framework Analysis approach^33^ in line with methods used in our larger study^2^ (Appendix III). This initial analysis, (conducted by SD and JL and feedback from LJ), revealed participants’ limited understanding of their illness, and difficulties communicating with doctors about their disease, treatment and future prognosis. Patients’ perspectives on what appeared to be examples of medical power present in health care encounters also emerged.

A secondary data analysis using medical power as the framework was conducted by SD and JL (senior research health psychologist with 20 years of qualitative methods experience), with support from a medical sociologist (PH). As communication had emerged as the underpinning concept, we focused specifically on three types of patient-doctor interaction: (1) information exchange (2) decision-making (3) participants’ understanding of prognosis and future treatment plans. We extracted the relevant text from each transcript and conducted an analysis using a framework proposed by Sandelowski & Leeman (2012)^34^ of translating findings into thematic sentences. Thematic sentences aim to summarise key ideas while preserving the complexity of concepts which have been identified. We used these thematic sentences as section headers, and provided supportive evidence from the transcripts. Working independently, SD and JL analysed the original data inductively, using medical power as the lens through which to interpret the data. JL and SD then met PH to review the analysis. We discussed the appropriateness of the thematic sentences generated with LJ (Head of academic palliative care unit) and JW (consultant nurse in palliative care) to agree a consensus.

### Ethical approval

We sought ethical approval from the NRES Committee London – West London & GTAC (ref 14/LO/0799), but this committee deemed it unnecessary for the larger study which included the patient data. We obtained NHS permission from the Royal Free London Clinical Governance Lead for Hepatology and Palliative Care under the remit of health service improvement.

## Findings

### Background to clinical setting and participants’ schedules of care

Our sample of 10 people with cirrhosis included 5 who were on the liver transplant list and 5 who were not due to frailty, complications or continued alcohol misuse. All were reviewed in out-patient clinics by doctors of varying seniority from the liver team. Treatments and the management of care differed between the transplant and non-transplant patients. The decision to place a person on the liver transplant waiting list was made by clinicians, using internationally agreed clinical criteria.

Those eligible for a liver transplant regularly attended clinics every 4-6 weeks for intensive monitoring to check that they were eligible to remain on the waiting list They were reviewed by a member of the liver team and other healthcare specialists in the transplant team. Participants not eligible for a liver transplant attended clinic less frequently, and were monitored less intensively, but on occasions received active treatment to manage any complications of their cirrhosis.

### Emergent themes

We identified three concepts that explained doctor-patient communication from the perspective of our participants: (1) Patients want to portray a positive image of themselves; (2) Patients want to avoid being confrontational with doctors; (3) Patients feel powerless in the face of doctors’ superior medical knowledge. These concepts suggested deeper dynamic issues of power during medical encounters, illustrated by participants’ unwillingness to voice their concerns and express themselves, challenge decisions, or seek information; they may be intent on not upsetting the *status quo* and wish to present an optimal picture of themselves to secure the best treatment.

## 1) Patients want to portray a positive image of themselves

In the exchange of information during consultations, participants appreciated the input provided by doctors. Those eligible for a liver transplant saw transplantation as their life-saving option and recognised their dependence on their doctors for access for a transplant. They were keen to present themselves as being compliant and not questioning the doctor's advice, to be seen as grateful and did not wish to be viewed negatively. As a result, participants often did not complain or voice their concerns. This is illustrated by the following participant who wanted to discuss his concerns during a clinic appointment but felt unable to do so because doctors were pressed for time to meet their own work schedule.

“*The hospital is obviously exceptional it's a national centre of excellence for this type of disease and transplantation, so I feel happy that I’m a patient here I don't want to upset the apple cart, I’m not actually mistrustful of doctors at all, I do trust them and I put my faith in them, but my concerns, at my last meeting here, they were just driven out by the routine, before I knew it I was being ushered out the door despite having concerns.” Participant J (transplant list)*

Patients did not want to cause problems as demonstrated by this participant who developed distressing physical symptoms between her transplant clinic appointments. She purposefully did not mention what had happened at her next appointment for fear of being seen as troublesome.

“*At the moment I am having a lot of confusion, I’m hoping it is down to the disease in a way. When I came to my appointment I didn't mention it, you don't want to rock the boat, you don't want to come in and have a go, you’ve got to see these people every time, and they are responsible for your life.” Participant F (transplant list)*

In aiming to maintain a positive image of themselves in the eyes of the doctors, some participants did not active seek to ask further questions about their condition, although they had concerns. One participant had a fear his condition was more serious, but he was not being informed about his current medical status. He did not directly ask the doctor, or try to find out information about his prognosis from other members of staff, he only sought information about possible treatments. He had been given no information about his prognosis and would have liked to know more about its impact, but felt unsure whether this was something he could ask in clinic.

“*It must be life restricting in some way? I suppose it is actually up to me to ask, I presume?” Participant H (not on transplant list)*

## 2) Patients want to avoid being confrontational with doctors

Despite asking for information, participants often struggled to receive it. One participant was anxious to find out about the severity of their condition at their last appointment with their consultant, seeking more definitive answers from the doctor, who in turn was reluctant to address these questions until further tests were conducted. Doctors may find it challenging to discuss questions about prognosis, but it was not clear to this participant why the topic was not discussed and why he needed to do another test

“*Well, I was expecting more tests, I’d rather have that whether they’re good or bad and know what I am facing. I’ve always wanted to know, and I’ve always been honest about my drinking otherwise I’m lying to myself ….I asked Dr (surname) because you know you could die any moment, and obviously drinking has shortened my life expectancy, but I just wanted to, I asked him and he said we’ll do a blood test and that, and he got the results and there was another test he wanted to do, so I’m seeing him in 2 months’ time.” Participant A (not on transplant list)*

Some participants worried about the reasons doctors delayed giving them information or provided incomplete or conflicting information, illustrated by this participant who would have preferred to speak to the consultant (seen as the expert) about his medical condition and to have all his questions answered upon discharge from hospital. This participant's frustration is further enhanced by having to speak to the registrars (instead of the consultant), who between them gave conflicting information about his medical condition. Despite not seeing the consultant, this participant kept his frustration to himself.

“*I think a great one would have been to see the consultant before I was discharged …so he could actually tell me what was going on rather than one registrar telling me something and another registrar telling me something else and you know it's confusing. Someone who could actually tell me what was going on and who could answer really any questions and stuff, rather than being told that they’d contact me.” Participant I (not on transplant list)*

Participants not eligible for a liver transplant often took a passive role in decisions that affected them, expressing feelings of being overwhelmed by the complexity of their illness and fearful of making the wrong decisions. People generally wanted more information but lacked confidence to ask for clarification. Decisions on treatment were deferred to the judgement of doctors as people did not feel confident to make treatment decisions and trusted the doctors implicitly to act in their best interests. Sometimes people wanted extra time to discuss matters with others before making decisions but felt unable to request a delay in decision-making as this might inconvenience the doctors.

One participant had difficulties deciding whether or not to have an invasive procedure called a Transjugular Intrahepatic Portosystemic Shunt (TIPS). She was aware the doctors were busy and believed that deliberating over a decision would inconvenience them, so she suppressed her needs.

“*They suggested I have a TIPS that was what I needed. I wanted to discuss it with Dr XX, but they didn't want to delay too much. I thought there is no point in wasting Dr XX's time, so I decided to go ahead with it.” Participant B (not on transplant list)*

## 3) Patients feel powerless in the face of doctors’ superior medical knowledge

In the context of medical power, participants expressed powerlessness in three ways: (1) limited understanding of the medical terminology used; (2) limited understanding on the hierarchy of how health care is delivered; (3) doctors taking control without patient consent. Ultimately these factors influence how decisions are made during doctor-patient interaction and explain why participants had a lack of understanding of both their disease and the treatment options available to them.

### Patients’ limited understanding of medical terminology

For all participants (whether eligible or ineligible for a transplant), medical power is manifest in the focus of the consultation and the language used. Participants perceived that doctors’ focus of communication centred on the technical aspects of care such as specific diagnostic tests, medication or treatments. Participants revealed some frustrations as they wanted to express how liver disease was affecting them. One participant who experienced burdensome symptoms, had difficulties expressing this to his doctor.

“*There was nobody really that I felt comfortable with that I could divulge that sort of information to. You don't know if you’re going ‘mad’ at the end of the day for want of a better phrase.” Participant I (not on transplant list)*

Participants found it difficult to understand the language used by doctors which in turn affected their understanding of information received. They were often confused about the significance of what some of the information they received actually meant in terms of their health. As one participant commented:

“*They are talking in big long medical words; I mean I haven't read the medical dictionary so I’m not going to know.” Participant G (transplant list) *

This participant then continued that if he did not understand the medical language, the doctors were able to explain things to him in lay person's terms when required, demonstrating it was the doctors’ preference to use medical language in the first instance when talking about liver disease.

### Patients’ lack of understanding of the hierarchy of health care

Participants did not understand that the decision to place someone on the transplant list was taken amongst an implicit hierarchy of clinicians at a multi-disciplinary meeting (MDT) based on specific guidelines, medical evidence and clinical expertise. Participants were not invited to these meetings and lacked control in this situation, and remained unable to influence decisions. One participant expressed his powerlessness regarding the decision to be accepted onto the transplant waiting list:

“*I have been told they have discussed my situation at one of those MDT meetings. I don't understand what goes on at those meetings. I only receive some of the information from doctors.”*

*“There is a degree of helplessness here. I am in other people's hands as regards my health.” Participant J (transplant list)*

Although many participants tended to perceive that their doctors controlled the process of selection to the transplant waiting list, in reality, doctors have to adhere to strict guidelines and clinical indicators to help them determine whether a patient is suitable. This process is not transparent to participants who believe that doctors have more control to make decisions in multi-disciplinary meetings about transplant suitability**.** One participant was asked by his liver doctor whether he wanted to have his name put forward for the transplant list Given his perceived self-stigma he expressed reservations, as he did not feel he was a worthy candidate due to his previous alcohol misuse.

“*I’m not going to have a liver transplant because I’ve done this to myself and somebody else deserves the chance.” Participant C (transplant list)*

### Participant C (transplant list)

Generally, people found weighing up detailed clinical information and associated risks which they did not always understand very difficult. The following participant was anxious about making a decision about a TIPS procedure.

“*The doctor said some people get encephalopathy. I’ve got to have an EEG (Electroencephalogram) and that worries me a bit that it can affect your brain; that does worry me but it's a low percentage of encephalopathy. It worries me about having a TIPS because I like to drive so I want to, but the doctor said that that's ok….this side of TIPS does worry me, I must admit.*” *Participant H (not on transplant list)*

In cases where participants are temporarily incapacitated due to a medical emergency, doctors have to make decisions in what they believe is their patients’ best interests, in liaison with those important to the patient and legitimised by law, a form of medical paternalism. One participant understood that in some situations, doctors needed to take control of the medical care, but was angered that following a hospital admission for complications and temporary incapacity, she was not subsequently consulted by her doctors when she regained capacity, about further decisions to do with her care.

“*This is the thing you know, everyone expects you to do what they think, rather than what you want to do…I mean when you’re in the hospital bays you have to go along with what goes on, but it gets on your nerves a bit sometimes….I know they mean well……but you’re supposed to just knuckle down and do what they say.” Participant D (not on transplant list) *

This sense of powerlessness meant that participants felt that doctors often appeared to control the information given and implicitly decided the extent and depth of frank discussions about prognosis and medical status. This may have been a well-meaning attempt to preserve a sense of hope or stem from doctors’ internal struggles to provide the best for their patients, but it may have continued to perpetuate an unequal power differential. Many participants wanted both more information about how their condition was likely to progress and possible treatment options. However, they were often unsure how to gain this information from their doctors.

Participants on the liver transplant waiting list felt that doctors did not engage in conversations about how soon a transplant might become available or where they ranked on the list Whilst this would be dependent on the availability of suitable donors, participants felt that doctors were unwilling to discuss possible prognosis should they be unable to have a transplant.

“*I’d like to know what the general prognosis will be if I don't …. If I’m not going to get a liver for example, and there is no way of knowing if I am going to get a liver or not, if I’m not, I wouldn't mind knowing what the general prognosis will be with someone in my condition……, whether the cirrhosis will do for me first or whether the (liver) cancer will spread first!” Participant J (transplant list)*

Some participants understood it was difficult for doctors to predict the future even if a donor did become available. They perceived that doctors relied on medical statistics when imparting information about transplants. This use of unfamiliar referents appeared to buttress their superior knowledge and training. At the same time, doctors occasionally used more direct language when they discussed medical risks, suggesting that when necessary they were able to speak on a participants’ level of understanding.

Amongst those not on the transplant list there was a lack of awareness about whether their condition was amenable to cure and what optimal symptoms management could potentially achieve. Treatment plans were not generally challenged by participants. One participant was particularly anxious that no further treatment plans were put in place.

*“I would really like to know whether, well really what more can be done”.* *…..it's a bit concerning, I would like a plan or a course of action because you like to see things progressing and I seem to be in a bit of limbo at the moment.”* *Participant I (not on transplant list)*

As a result of this perceived control in the flow of information, some participants expressed how difficult it was for them to manage the lack of information about possible future care. For some, the result was a lack of understanding about the status of their disease as they feared they were not in possession of all the medical facts and were suspicious that their condition was more serious than they initially thought. During a hospital admission a participant questioned his medical status based on where his bed was situated on the ward.

“ *it would have been nice to be in one of the side wards with about 4/5 beds in, with people who were like myself hopefully recovering, rather than with people who are expecting to be dead shortly, ………because you’re thinking that could be me next time…… or is that going to happen to me?” Participant I (not on the transplant list)*

## Discussion

Our findings illustrate the complexity of doctor-patient interactions in a specialist centre. Many people with cirrhosis allude to concepts reflecting medical paternalism and some deliberately change their engagement with doctors to maximise their perceived chances of positive health care outcomes. Some appear to believe that in controlling transplant opportunities doctors hold power over life and death. Given the historical power dynamics in doctor-patient relations,^35^ people with cirrhosis are less able to play a proactive role in determining the information they receive and the implications of this knowledge. It is this dimension of implicit power to which we draw attention.

Our participants perceived that doctors shape patients’ responses to their illness by: not sharing information in a way they can understand; not appearing to seek and value patient opinion; sometimes making decisions on their behalf; inadvertently encouraging dependency. Our participants were fearful both of the seriousness of their illness and its implications, but were frightened of seeking further information from doctors, as they saw them as important and powerful and feared seeming inadequate. Our participants felt compelled to conform to socially acceptable behaviours to present an optimal picture of themselves and be considered worthy recipients of healthcare. The doctor-patient interaction is held in high regard by patients as they feel they must maintain this as a constructive relationship,^35^ based on trust and in accordance the wider social order.^36^ Patients are often viewed by doctors as grateful recipients of care and people with cirrhosis are rarely treated as equals. Contributing to this imbalance are existential fear created by the illness, and an indirect fear of doctors whom people with cirrhosis consider to be all powerful.^37^ Patients often display poor understanding of cirrhosis and lack opportunities for self-determination, being unable to articulate their concerns, or challenge their doctors.^38^ Healthcare encounters are characterised by patients’ passivity, settling on a dependent relationship which may partly be explained by patients’ perceived self-stigma.^10^ Once participants are unable to express themselves within the doctor-patient interactions their voice and agency become limited.^36^

We do not suggest that this disempowerment is a result of deliberate action by doctors. It is more likely a complex interaction of time pressures, economic stringency with regard to the costs of expensive treatments such as transplants, and lack of training and confidence in raising issues concerning the approach of end of life.^11,12^

Our findings have several implications for clinical practice. Doctors could be encouraged and supported to allow patients to ask difficult questions or express wishes on treatments;^39^ failure in this might be described as palliative paternalism, where doctors restrict themselves by using questions with concrete options during their consultations.^40^ It is possible to work with doctors to help them pick up patient cues, for example using shared care approaches and joint clinics with palliative care specialists who are well-trained in facilitating difficult conversations. Doctors need to give information to patients so that they can understand, and comprehension of the information should be checked to clarify any misunderstandings. Doctors also need to recognise and accept their own limitations and powerlessness to cure,^41^ placing less emphasis on hope and faith in technology.^42^

Our aim in exposing these issues is to promote constructive discussion of doctor-patient interactions in advanced liver disease, to enhance the experience of both patients and clinicians and enable more satisfactory outcomes. A comprehensive screening of patient’s symptoms rather than relying on self-report might uncover how patients are really suffering and open up treatment options. Patients could be involved in the co-design of hepatology services to encourage them not to self-limit or self-judge.

## Limitations and implications for future research

Our findings focused only on the patient perspective and a deeper understanding would have been gained by exploring the perspectives of clinicians. The study was conducted in one liver transplant unit with a small sample size, which may reduce the generalisability of the findings. There is potential bias in our patient recruitment; although we purposively selected our participants for key characteristics, our small sample size may mean that we have not captured the full range of experiences, and we did not include participants from diverse ethnic backgrounds. Ethnography is a preferable method to capture non-verbal behaviours in medical encounters. More research using ethnographic approaches in different healthcare settings involving tertiary non-liver transplant units and liver teams in less specialised secondary care for people with liver disease would enhance our understanding of clinician- patient interactions in cirrhosis.

## Conclusion

The persistence of power relations remains evident during clinical encounters in liver disease. Given the large investment in creating more equal relationships in health care and increasing shared decision-making, it is salutary to be reminded that older, more hierarchical relationships still persist and may can have unintended negative implications for patients. More balanced relationships may improve the experience of illness for those with cirrhosis and allow for more reflective practice amongst clinicians that may enhance their working lives.

## Topic guide: Interviews with patients with advanced liver disease

**Diagnosis**
  - When did you first receive your diagnosis?
  - What were you diagnosed with?
**Understanding**
  - What were you initially told about your diagnosis?
  - What is your understanding of your diagnosis now?
**Symptoms**
  - Have you had any physical symptoms?
  - Have you had any psychological symptoms?
**Services and support**
  - Did/do you see your GP for help and advice about your condition?
  - Who provides medical care for your condition now?
  - How often do you receive care for your condition?
  - What type of care do you receive?
**Prognosis and future**
  - Do the doctors/nurses discuss how your condition is likely to progress?
  - What do you worry about for the future?
  - Have you discussed this with your medical team?
**Improvements in care**
  - Do you have any specific worries about your care?
  - How do you think your care could be improved?

## Standards for Reporting Qualitative Research (SRQR)*

http://www.equator-network.org/reporting-guidelines/srqr/

**Table:**

		**Page/line no(s).**
**Title and abstract**		
	**Title** - Concise description of the nature and topic of the study Identifying the study as qualitative or indicating the approach (e.g., ethnography, grounded theory) or data collection methods (e.g., interview, focus group) is recommended	P1, l.2-3
	**Abstract** - Summary of key elements of the study using the abstract format of the intended publication; typically includes background, purpose, methods, results, and conclusions	P2, l.32-49
		
**Introduction**		
	**Problem formulation** - Description and significance of the problem/phenomenon studied; review of relevant theory and empirical work; problem statement	P3-6, l. 57-133
	**Purpose or research questio**n - Purpose of the study and specific objectives or questions	P6, l.134-135
		
**Methods**		
	**Qualitative approach and research paradigm** - Qualitative approach (e.g., ethnography, grounded theory, case study, phenomenology, narrative research) and guiding theory if appropriate; identifying the research paradigm (e.g., postpositivist, constructivist/ interpretivist) is also recommended; rationale**	P6, l.139-141.
	**Researcher characteristics and reflexivity** - Researchers’ characteristics that may influence the research, including personal attributes, qualifications/experience, relationship with participants, assumptions, and/or presuppositions; potential or actual interaction between researchers’ characteristics and the research questions, approach, methods, results, and/or transferability	P6, l.148; P8, l.176-177,
	**Context** - Setting/site and salient contextual factors; rationale**	P6, l.153-154.
	**Sampling strategy** - How and why research participants, documents, or events were selected; criteria for deciding when no further sampling was necessary (e.g., sampling saturation); rationale**	P7, l.152-157.
	**Ethical issues pertaining to human subjects** - Documentation of approval by an appropriate ethics review board and participant consent, or explanation for lack thereof; other confidentiality and data security issues	P6, l.149-151. P8, l.191-195.
	**Data collection methods** - Types of data collected; details of data collection procedures including (as appropriate) start and stop dates of data collection and analysis, iterative process, triangulation of sources/methods, and modification of procedures in response to evolving study findings; rationale**	P8, l.191-195.
	**Data collection instruments and technologies** - Description of instruments (e.g., interview guides, questionnaires) and devices (e.g., audio recorders) used for data collection; if/how the instrument(s) changed over the course of the study	P7, l.164. Appendix I
	**Units of study** - Number and relevant characteristics of participants, documents, or events included in the study; level of participation (could be reported in results)	P7, 1.153-157.
	**Data processing** - Methods for processing data prior to and during analysis, including transcription, data entry, data management and security, verification of data integrity, data coding, and anonymization/de-identification of excerpts	P7, l.173-174.
	**Data analysis** - Process by which inferences, themes, etc., were identified and developed, including the researchers involved in data analysis; usually references a specific paradigm or approach; rationale**	P7, l.169-188.
	**Techniques to enhance trustworthiness** - Techniques to enhance trustworthiness and credibility of data analysis (e.g., member checking, audit trail, triangulation); rationale**	P8, l.185-188.
		
**Results/findings**		
	**Synthesis and interpretation** - Main findings (e.g., interpretations, inferences, and themes); might include development of a theory or model, or integration with prior research or theory	P9 -18, l.214 -420.
	**Links to empirical data** - Evidence (e.g., quotes, field notes, text excerpts, photographs) to substantiate analytic findings	P9-18, l.214 –420.
		
**Discussion**		
	**Integration with prior work, implications, transferability, and contribution(s) to the field -** Short summary of main findings; explanation of how findings and conclusions connect to, support, elaborate on, or challenge conclusions of earlier scholarship; discussion of scope of application/generalizability; identification of unique contribution(s) to scholarship in a discipline or field	P18-20, l,422 – l.467.
	**Limitations** - Trustworthiness and limitations of findings	P20, l.470-480.
		
**Other**		
	**Conflicts of interest** - Potential sources of influence or perceived influence on study conduct and conclusions; how these were managed	P28, l. 636
	**Funding** - Sources of funding and other support; role of funders in data collection, interpretation, and reporting	P28, l.639-645
		
	*The authors created the SRQR by searching the literature to identify guidelines, reporting standards, and critical appraisal criteria for qualitative research; reviewing the reference lists of retrieved sources; and contacting experts to gain feedback. The SRQR aims to improve the transparency of all aspects of qualitative research by providing clear standards for reporting qualitative research.	
	**The rationale should briefly discuss the justification for choosing that theory, approach, method, or technique rather than other options available, the assumptions and limitations implicit in those choices, and how those choices influence study conclusions and transferability. As appropriate, the rationale for several items might be discussed together.	

**Reference:** O'Brien BC, Harris IB, Beckman TJ, Reed DA, Cook DA. **Standards for reporting qualitative research: a synthesis of recommendations.** *Academic Medicine*, Vol. 89, No. 9 / Sept 2014 DOI: 10.1097/ACM.0000000000000388

## Appendix : Summary of the Framework Analysis used in the initial data analysis

SD first transcribed all interviews verbatim, after which both SD and JL (lead investigator) read through all the transcripts independently to become familiarized with the data. In re-reading the transcripts line by line, both SD and JL identified codes from the data to describe what has been interpreted, a process known as indexing. After analysing the first four transcripts, we developed a working analytic framework by grouping the codes identified into clearly defined categories after consultation with the rest of the research team. We then applied the analytical framework to the remaining transcripts, using an Excel spreadsheet to generate the framework matrix. This involved summarizing data by category from each transcript, a process known as ‘charting’, where data was rearranged to allow easy comparison, both within and between participants. In our final stage, we conducted a thematic analysis (Braun and Clarke) on this data to identify key themes. To ensure validity and reliability, the two researchers (SD, JL) independently carried out theme generation, who later met to discuss the findings.
